# Multi-Omics Analysis of Novel Signature for Immunotherapy Response and Tumor Microenvironment Regulation Patterns in Urothelial Cancer

**DOI:** 10.3389/fcell.2021.764125

**Published:** 2021-12-03

**Authors:** Guangdi Chu, Wenhong Shan, Xiaoyu Ji, Yonghua Wang, Haitao Niu

**Affiliations:** ^1^ Department of Urology, The Affiliated Hospital of Qingdao University, Qingdao, China; ^2^ Department of Nephrology, Qingdao Central Hospital, The Second Clinical Medical College of Qingdao University, Qingdao, China; ^3^ Department of Obstetrics and Gynecology, The Affiliated Hospital of Qingdao University, Qingdao, China

**Keywords:** tumor microenvironment, immunotherapy, urothelial cancer, macrophage, immune checkpoint

## Abstract

The tumor microenvironment (TME) is mainly composed of tumor cells, tumor-infiltrating immune cells, and stromal components. It plays an essential role in the prognosis and therapeutic response of patients. Nonetheless, the TME landscape of urothelial cancer (UC) has not been fully elucidated. In this study, we systematically analyzed several UC cohorts, and three types of TME patterns (stromal-activation subtype, immune-enriched subtype and immune-suppressive subtype) were defined. The tumor microenvironment signature (TMSig) was constructed by modified Lasso penalized regression. Patients were stratified into high- and low-TMSig score groups. The low-score group had a better prognosis (*p* < 0.0001), higher M1 macrophage infiltration (*p* < 0.01), better response to immunotherapy (*p* < 0.05), and more similar molecular characteristics to the luminal (differentiated) subtype. The accuracy of the TMSig for predicting the immunotherapy response was also verified in three independent cohorts. We highlighted that the TMSig is an effective predictor of patient prognosis and immunotherapy response. Quantitative evaluation of a single sample is valuable for us to combine histopathological and molecular characteristics to comprehensively evaluate the status of the patient. Targeted macrophage treatment has great potential for the individualized precision therapy of UC patients.

## Introduction

Urothelial carcinoma of the bladder comprises two disease entities with different molecular characteristics and clinical outcomes (Knowles and Hurst, 2015). It is one of the most common malignant tumors of the genitourinary system, and it was estimated that there will be 83,730 new cases and 17,200 deaths worldwide in 2021 (Siegel et al., 2021). Non-muscle invasive bladder cancer (NMIBC) accounts for approximately 70% of newly diagnosed bladder cancers and comprises different entities, including carcinoma *in situ* (CIS), noninvasive nipple tumors, and papillary tumors invading the lamina propria. The overall survival (OS) rate of patients with NMIBC has been approximately 90% for 5 years. However, approximately 15 to 20% of NMIBC progresses to muscle invasive bladder cancer (MIBC), and CIS and advanced papillomas are more likely to progress to MIBC than low-grade papillomas. MIBC refers to tumor invasion of the detrusor, the prognosis of it is poor. It easily metastasizes and the determination of treatment is complex and difficult (Knowles and Hurst, 2015; Dinney et al., 2004; Kamat et al., 2016; Siegel et al., 2018; Magers et al., 2019; Patel et al., 2020).

Over the past few decades, cancer treatment has undergone revolutionary changes, from traditional chemotherapy and radiation-targeting of tumors to antibody-based immunotherapy. This antibody-based therapy can more accurately regulate the immune response to tumors. The clinical treatment for metastatic urothelial carcinoma has also changed dramatically due to recent immunotherapy developments (Li et al., 2019a; Powles et al., 2020). Immunotherapy with immune checkpoint blockades, such as those targeting PD-1/PD-L1 and CTLA-4, has shown amazing clinical benefit in a small number of patients who achieve a persistent response. However, the clinical efficacy in most patients is small or nonexistent, far from meeting clinical needs (Topalian et al., 2012).

Traditional cognition holds that tumor progression is caused only by alterations in the genetic or epigenetic characteristics of tumor cells. However, with the gradual deepening of research, it has become clear that the TME also plays a key role in the growth and survival of tumor cells (Zhang et al., 2020a). Tumor cells can not only adapt and survive in such environments but also evade the detection and elimination by the host immune surveillance system by disguising themselves as normal cells. It can also induce various biological behavior changes by directly and indirectly interacting with other TME components, inducing processes such as cell proliferation, immune tolerance, and angiogenesis (Zhang et al., 2020a; Zhang et al., 2020b). Determining the status of TME at the time of diagnosis can help determine their response to immunotherapy (Rosenberg et al., 2016) and provide information on the benefit of chemotherapy (Jiang et al., 2018). The changes in the infiltration levels of CD8^+^ T cells, CD4^+^ T cells and tumor-associated macrophages in the TME are related to the prognosis of a variety of malignant tumors, including urothelial carcinoma, melanoma, lung cancer, breast cancer and gastric cancer (Turley et al., 2015; Nishino et al., 2017; Mariathasan et al., 2018; Zeng et al., 2019). Increasing evidence has confirmed the clinicopathological significance of TME infiltration for predicting patient prognosis and therapeutic responses. However, the comprehensive landscape of the TME in UC has not been fully elucidated up to now.

In this study, we comprehensively evaluated the TME pattern by integrating multi-omics data from multiple cohorts. The TME phenotype was associated with the genomic, clinical, and pathological features of UC and a scoring scheme was established to quantify the immune status of a single sample. The TMSig was constructed by modified Lasso penalized regression and could serve as a robust predictor of patient prognosis and immunotherapy response.

## Materials and Methods

We used five urothelial cancer cohorts were used in this study, including the IMvigor210 cohort (Balar et al., 2017), the TCGA-BLCA cohort (Robertson et al., 2017), the GSE32548 cohort (Lindgren et al., 2012), the GSE48075 cohort (Choi et al., 2014), and the UTUC cohort (Su et al., 2021). The TMSig constructed in the current study was assessed for prognostic ability in all five independent cohorts and the combined cohort. We also obtained pretreatment tumor expression profiles from three cohorts receiving immunotherapy to examine the response to immunotherapy in high- and low-scoring populations. Expression profile data for human cancer cell lines (CCL) data was from the Broad Institute Cancer Cell Line Encyclopedia (CCLE) (Ghandi et al., 2019). In addition, molecular and drug sensitivity data from two pharmacogenomic datasets (CTRP and PRISM) (Basu et al., 2013; Yu et al., 2016) of hundreds of CCLs were used to estimate drug response in clinical samples.

Additional detailed methodological descriptions, including the data preprocessing process, assessment of immune cell infiltration levels, identification of TME regulatory patterns, biofunctional analysis, TMSig construction process and evaluation of clinical applicability, clinical cohort drug sensitivity assessment, and statistical analysis were described in detail in Supplementary Materials and Methods.

## Results

### The Landscape of TME Immune Cell Infiltration of Urothelial Cancer and the Identification of TME Patterns

**FIGURE 1:**
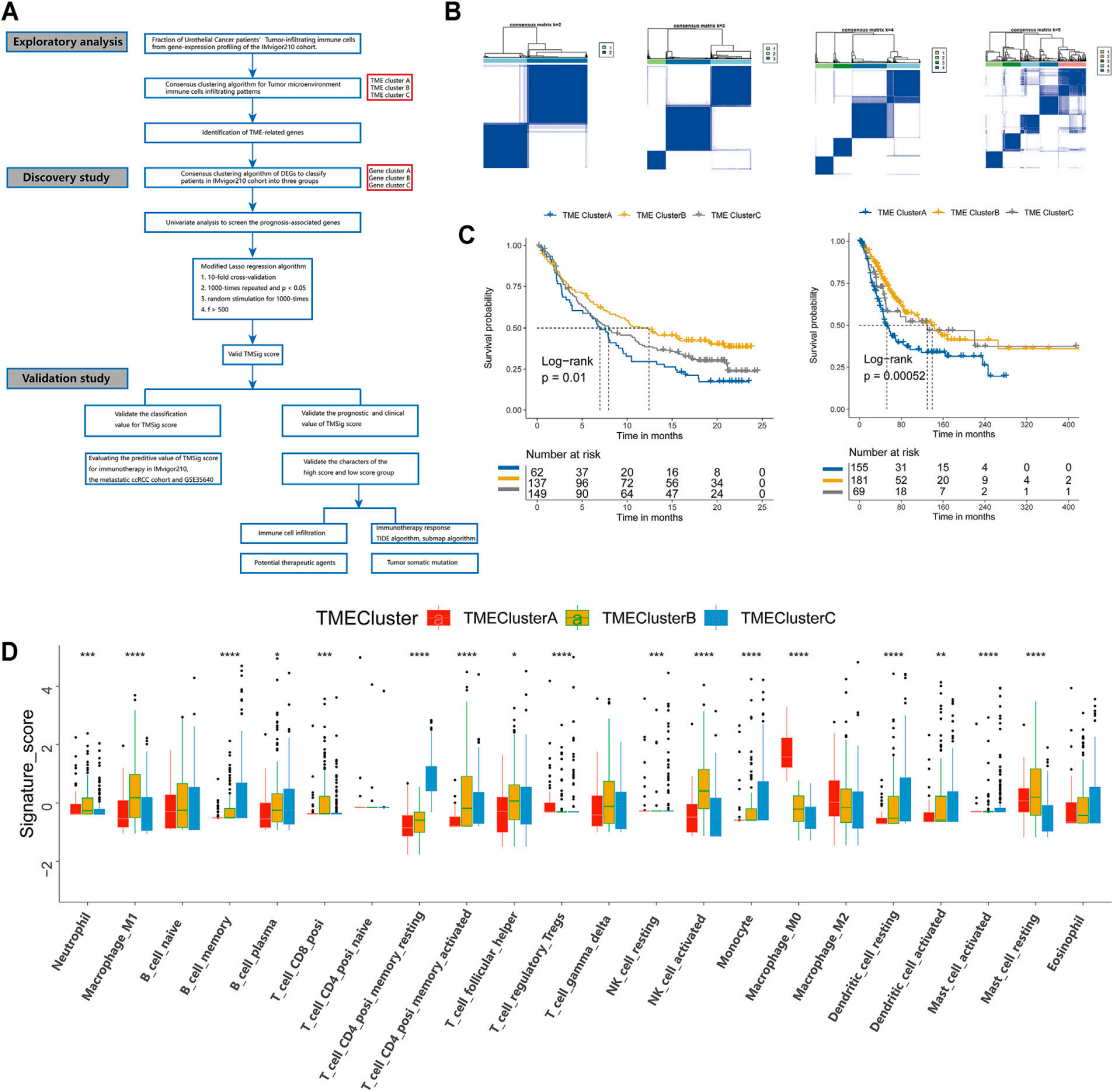
Landscape of the TME in urothelial cancer and characteristics of TME subtypes. **(A)** The overview of study design. **(B)** Consensus matrixes of all patients with urothelial cancer for each k (k = 2–5), displaying the clustering stability using 1,000 iterations of hierarchical clustering. **(C)** Kaplan-Meier curves for overall survival (OS) of urothelial cancer patients from the IMvigor210 cohort with the TME patterns. **(D)** Immune infiltration cells expressed in TMEclusters. The range of *p* values are labeled above each boxplot with asterisks (*p < 0.05, **p < 0.01, ***p < 0.001, ****p < 0.0001). **(E)** Kaplan Meier curves for overall survival (OS) of urothelial cancer patients from the TCGA cohort with the TME patterns.

An overview of our research is shown in Figure 1A. First, we systematically constructed a landscape of the TME immune cell network that comprehensively demonstrated the interactions between immune cells (Supplementary Figure S1A). Then, CIBERSORT algorithms were performed to quantify the infiltration levels of immune cells in UC tissues (Supplementary Table S1). According to the immune cell infiltration data and clinical information of 348 patients (Supplementary Table S2), we performed unsupervised clustering to classify the UC patients into three distinct subtypes (Figure 1B), including 62 patients in TME-ClusterA, 137 patients in TME-ClusterB, and 149 patients in TME-ClusterC (Supplementary Figure S1B). And there were significant differences in prognosis outcomes among these clusters. The TME-ClusterB exhibited a prominent survival advantage, while the prognosis of patients in TME-ClusterA was the worst (log-rank test, *p* = 0.01, Figure 1C). And the distribution of immune cell infiltration in the IMvigor210 cohort was shown in Figure 1D. In addition, we also performed CIBERSORT analysis in The Cancer Genome Atlas (TCGA) cohort and used the same parameters for consistent clustering. We found that the TCGA cohort could also be divided into three categories, and also had a significant difference in prognosis among three categories (log-rank test, *p* = 0.00052, Figure 1E), which further indicates the rationality of stratifying urothelial cancer patients according to TME characteristics. Interestingly, we found that in the TCGA cohort, there was a partial overlap in the survival curves between ClusterB and ClusterC, which is most likely due to a batch effect between these two cohorts. And the main conclusion that ClusterB has the best prognosis and ClusterA has the worst prognosis obtained by clustering is not affected.

**FIGURE 2:**
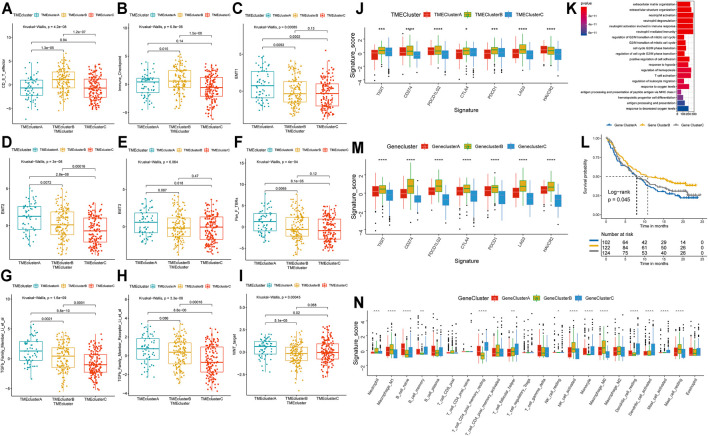
Molecular characterization of TME-Clusters and identification of Gene-Cluster. **(A)** CD8^+^T cell effector, **(B)** Immune checkpoint signature, **(C–E)** EMT-related signature, **(F)**. Pan_F_TBRs signature, **(G)** TGFb Family Member signature, **(H)** TGFb Family Member Receptor signature, **(I)** WNT target signature. **(J)** Immune checkpoints expression in TME-Clusters. **(K)** GO enrichment analysis of the TME-related genes. **(L)** Kaplan–Meier curves for Gene-Clusters. **(M)** Immune checkpoints expression in Gene-Clusters. **(N)** Immune infiltration cells expressed in Gene-Clusters. The range of *p* values are labeled above each boxplot with asterisks (*p < 0.05, **p < 0.01, ***p < 0.001, ****p < 0.0001).

Immune-associated cells could reflect the characteristics of individual immune microenvironment to a certain extent, and the immune checkpoint is also considered to be an important factor in predicting the response to immunotherapy. The Kaplan-Meier analysis we performed also showed that patients with different levels of immune cell infiltration and immune checkpoint expression had significant difference in clinical prognosis (Supplementary Figure S2). In order to explore the characteristics of patients in different patterns, we carried out a detailed comparison of them. The expression levels of CD8^+^ effector T cells and immune checkpoints in patients of TME-ClusterB were higher than those in patients in the other clusters (*p* < 0.05) (Figures 2A,B). These results strongly imply that patients in TME-ClusterB may be more likely to benefit from immunotherapy, which is consistent with the favorable prognosis in TME-ClusterB patients. The high level of immune-associated cell infiltration level also indicated that this cluster may be associated with multiple immune-related responses or activities and could be identified as immune-enriched subtype. TME-ClusterA was associated with the activation of epithelial-mesenchymal transition (EMT), the transforming growth factor-β (TGF-β), and Wnt signaling pathways (Figures 2C–I). The expression of specific immune checkpoints was also lower in this cluster (Figure 2J). The patients in this cluster had the worst prognosis, and the infiltration levels of T regulatory cells and M0 and M2 macrophages in this cluster were significantly higher than those in other clusters. Based on these characteristics, this cluster could be identified as the stromal-activation subtype. Interestingly, we also observed abundant immune cell infiltration in TME-ClusterC, such as memory B cells, plasma cells, CD4^+^ memory resting T cells, monocytes, resting dendritic cells, activated dendritic cells, activated mast cells, and eosinophils, but the relative abundance of immune cells did not significantly change the prognosis of these patients, and their powerful antitumor effect was suppressed. So, we defined this group as the immune-suppressive subtype.

### Identification of TME-Cluster Related Differentially Expressed Genes (DEGs) and Functional Analysis

To clarify the unique biological role of each cluster in the TME, we performed a differential expression analysis with the limma package. Each cluster was compared with the other clusters in the cohort, and a total of 7,996 DEGs were identified (Supplementary Figure S3A and Supplementary Table S3). Based on these DEGs, the GSVA package was used to analyze the specific enrichment pathways of each cluster (Supplementary Table S4). We found that TME-ClusterA was significantly enriched in HALLMARK EPITHELIAL MESENCHYMAL TRANSITION, HALLMARK COAGULATION, and HALLMARK ANGIOGENESIS, which may be related to the poor prognosis outcome (Supplementary Figure S3B). We conducted a functional enrichment analysis by the clusterProfiler R package (Supplementary Table S5) and found enrichment mainly in neutrophil activation, neutrophil-mediated immunity, T cell activation, regulation of innate immune response, and other immune-related Gene Ontology (GO) terms (Figure 2K). This once again proved the close relationship between the DEGs and immune-related functions.

To further explore the association between the DEGs and phenotypes, we conducted another unsupervised clustering analysis (Supplementary Figure S3C) and found that the cohort could also be divided into three cohorts with significant prognosis differences (log-rank *p* = 0.045) (Figure 2L). We referred to these as Gene-Clusters A, B, and C. Patients in Gene-ClusterB had the best prognostic outcomes, while patients in Gene-ClusterA had the worst. Using a chi-square analysis to compare the gene clusters and TME patterns, we found good consistency between these two grouping methods (χ^2^ contingency tests, *p* < 2.2e-16). The distribution of patients by TME patterns and TME gene clusters are shown and specific information can be found in Supplementary Table S6. There were also significant differences in the level of immune checkpoint expression (Figure 2M) and immune cell infiltration (Figure 2N) among these gene clusters, indicating that these gene clusters could also represent the TME characteristics of the patients.

### Construction of the TMSig

**FIGURE 3:**
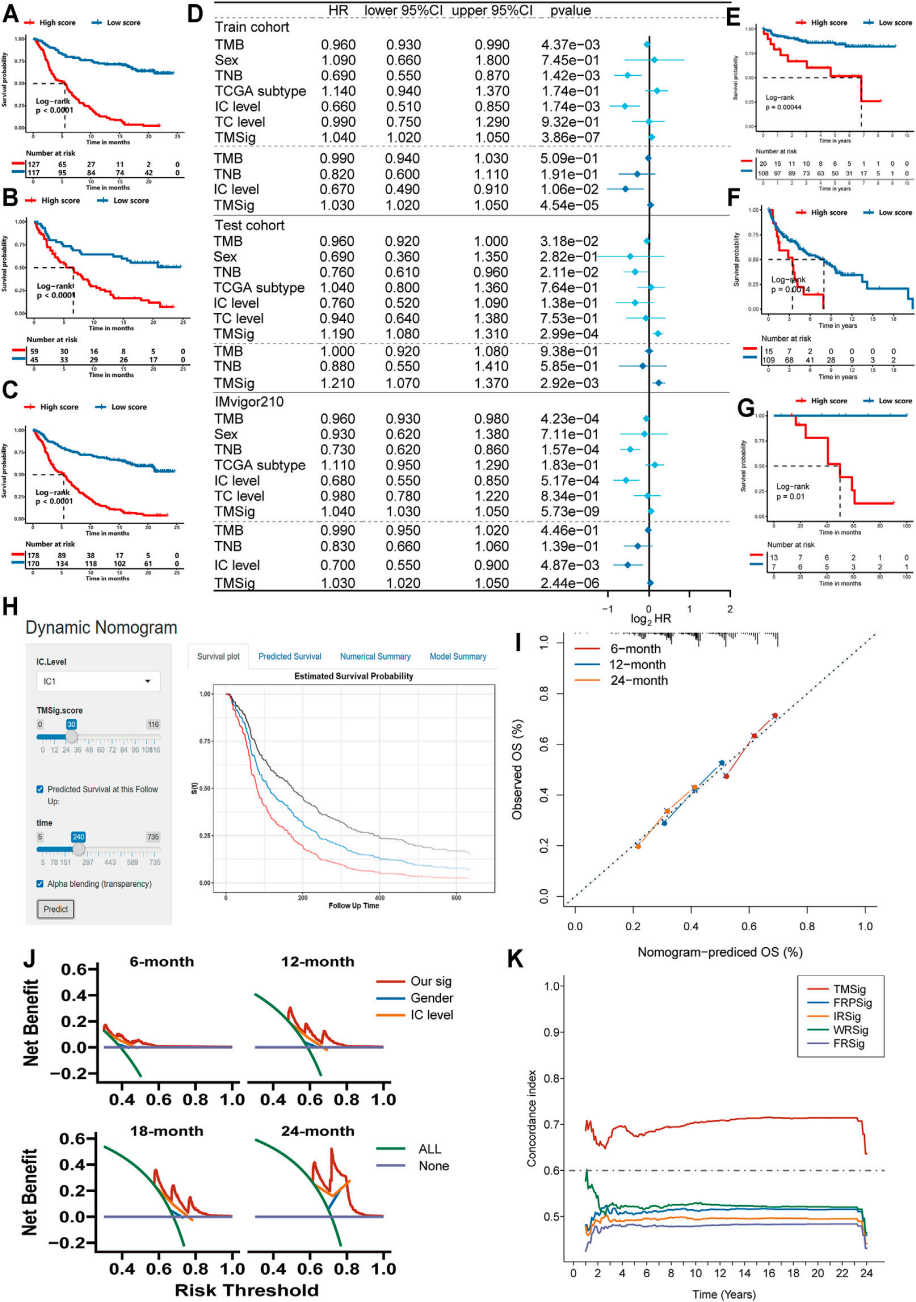
Exploring the clinical practice value of TMSig. **(A)** Kaplan-Meier curves for overall survival (OS) of patients from the train cohort. **(B)** Kaplan-Meier curves for OS of patients from the test cohort. **(C)** Kaplan-Meier curves for OS of patients from the IMvigor210 cohort. **(D)** Independent prognostic analysis of TMSig. **(E)** Kaplan-Meier curves for OS of patients from the GSE32548 cohort. **(F)** Kaplan-Meier curves for OS of patients from the GSE48075 cohort. **(G)** Kaplan-Meier curves for OS of patients from the UTUC cohort. **(H)** Dynamic nomogram for clinical practice. **(I)** Calibration curve analysis. **(J)** The decision curves analysis. **(K)** The c-index of TMSig compared with other signatures.

For the TME-related DEGs, we first matched expression data with clinical information, then reduced the dimension by using the univariate Cox regression model and used the more stringent *p* < 0.01 as the screening criterion to select 318 prognosis-related genes for further analysis (Supplementary Table S7). Next, we divided the IMvigor210 cohort into a training set and testing set at a ratio of 7:3. In the training set (*n* = 244), we performed modified Lasso regression analysis to construct the TMSig. In the process of cyclic calculation, we found that the maximum AUC value of the TMSig at 2 years was 0.906 (Supplementary Figure S4A). We defined the gene signature present at this time as the best candidate model. The prognosis of the low-score group was significantly better than that of the high-score group in the training set (log-rank *p* < 0.0001) (Figure 3A). Similar to the results obtained with the training set, the low-score group had a better prognosis in the internal testing set (log-rank *p* < 0.0001) (Figure 3B) and in the entire IMvigor210 cohort (log-rank *p* < 0.0001) (Figure 3C). The ROC curves proved the robust predictive ability of the TMSig, and the AUC at 1 year was 0.88 and 0.906 at 2 years in the training set. The AUC at 1 year was 0.747, and that at 2 years was 0.805 in the testing cohort. In the whole cohort, the AUC was 0.840 at 1 year and 0.876 at 2 years (Supplementary Figures S4B–D). Then we performed the univariate Cox regression algorithm to analyze TMSig together with other clinical characteristics of the patients in the training set. And further included them in the multivariate Cox regression algorithm after screening out the features with *p* < 0.05. And the same analysis was performed not only in the train cohort, but also in the test cohort and the entire IMvigor210 cohort. The *p*-value of TMSig < 0.05 in each time of analysis, proving that it can be served as an independent prognostic factor for patients (Figure 3D). And the TMSig also showed better prognostic predictive power in three independent cohorts (Figures 3E–G).

The TMSig score had a significantly different distribution in BOR, immune phenotype, IC level and other subgroups (Kruskal−Wallis, *p* < 0.05) and had a difference in TC level, but it was not significant (Supplementary Figures S4E–H). To further verify the reliability of the TMSig for predicting the prognostic outcomes, we performed a stratified analysis based on the clinical information of the IMvigor210 cohort. Through the Kaplan-Meier analysis, we found the TMSig has a great performance in several clinical subgroups (Immune phenotype: immune desert type, immune excluded type, immune inflamed type, IC level: IC0, IC1, IC2, Sex: Male, Female, BOR: SD/PD, CR/PR, TC level: TC0, TC1, TC2, Tobacco history: NEVER, PREVIOUS OR CURRENT, Supplementary Figures S4I–W). We also conducted an external verification of the prognostic value of the TMSig in the independent TCGA-BLCA cohort and found that it was of great significance for predicting both the overall survival (OS) and disease-specific survival (DSS) rates of these patients (Supplementary Figures S5A,B). In addition, stratified analysis of the TCGA cohort showed that the TMSig had significant prognostic significance in patients with higher disease stages (Supplementary Figures S5C,D), which inspired us to conclude that the TMSig may play a unique role in predicting the prognosis of patients with advanced neoplasia.

To improve the clinical application of TMSig, we constructed dynamic nomogram (TMSigDynNomapp: https://the-nomogram.shinyapps.io/TMSigDynNomapp/, Figure 3H), while calibration plots showed that comprehensive signature has accurate predictive power at different time points (Figure 3I). Decision curve analysis also showed that comprehensive signature can provide better clinical benefit to patients compared to applying gender, IC level, and other indicators for prediction (Figure 3J). Compared with other previously reported bladder cancer-related signatures, TMSig also has more robust predictive power (Figure 3K).

### The TMSig Could Effectively Predict Patient Response to Immunotherapy and Correlates With Immune Cell Infiltration, Tumor Mutation Load (TMB), and Tumor Neoantigen Burden (TNB)

We used ROC curves to evaluate the ability of the TMSig score to predict the efficacy of immunotherapy among patients in the IMvigor210 cohort and compared the score with known effective predictors such as TMB (Samstein et al., 2019), TNB (Wolf et al., 2019), and M1 macrophages (Zeng et al., 2020). It was found that the accuracy of the TMSig in effectively predicting the response to immunotherapy was not inferior to that of other biomarkers. (TMSig score AUC: 0.826, TMB AUC: 0.728, TNB AUC: 0.767, M1 macrophage AUC: 0.702) (Supplementary Figure S5E). To fully demonstrate the robustness of the TMSig for predicting immunotherapy response, we included two independent data sets for external validation. The AUC predicted by the TMSig score in the data from Miao et al. was 0.75 (Supplementary Figure S5F), and the AUC predicted by the TMSig score in the GSE35640 dataset was 0.687 (Supplementary Figure S5G). All these results indicate the great potential of the TMSig for discriminating patients who may benefit from immunotherapy.

**FIGURE 4:**
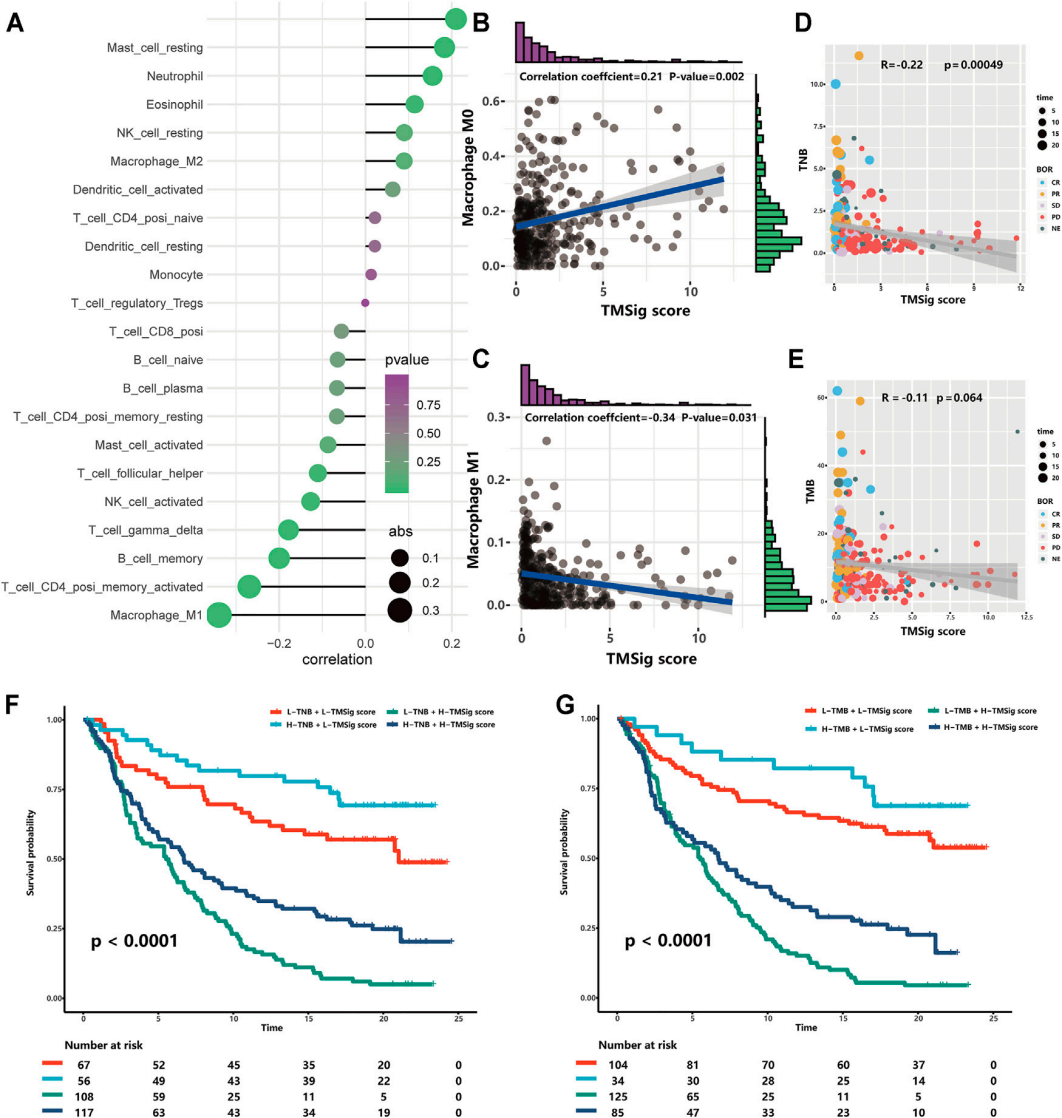
The association of TMSig score with immune-associated cells, TMB and TNB. **(A)** The correlation of TMSig score with immune cell infiltration level. The depth of the color represents the *p*-value and the size of the dot represents the absolute value of the correlation coefficient. **(B)** The correlation of TMSig score with M0 Macrophage. **(C)** The correlation of TMSig score with M1 Macrophage. **(D)** Modest but significant correlation of TMSig score with TNB. **(E)** The correlations of TMSig score with TMB. **(F)** Kaplan-Meier curves for patients stratified by both TNB and TMSig score. **(G)** Kaplan-Meier curves for patients stratified by both TMB and TMSig score.

It is well known that patients with different infiltration levels of immune cells have different prognostic outcomes or treatment responses. Therefore, we performed a Spearman correlation analysis to explore the relationship between the TMSig score and the infiltration level of various immune cells and found that there was a significant positive correlation between the TMSig score and M0 macrophages, resting mast cells, neutrophils, and eosinophils (*p* < 0.05, cor > 0) and a significant negative correlation with the infiltration level of follicular helper T cells, activated NK cells, gamma delta T cells, memory B cells, CD4^+^ memory-activated T cells, and M1 macrophages (*p* < 0.05, cor < 0) (Figure 4A). The strongest positive correlation was between the TMSig score and M0 macrophages (Figure 4B), and the strongest negative correlation was between the TMSig score and M1 macrophages (Figure 4C). There was a moderate but significant negative correlation between the TMSig score and TNB (Kruskal-Wallis, *p* = 0.00049) (Figure 4D), and the correlation between the TMSig score and TMB also demonstrated the same trend (Figure 4E). Intriguingly, combining the TMSig score with TMB or TNB contributed to the survival assessment (Kaplan-Meier analysis, TMSig score +TNB binary: *p* < 0.0001; TMSig score +TMB binary: *p* < 0.0001) (Figures 4F,G). We should clear that the correlation between TMSig and M1 macrophages is strong and deserves focused attention. But its correlation with TMB or TNB is moderate, which could provide direction for our study, but the exact relationship needs to be verified by further studies.

### Characteristic Differences Between the High and Low TMSig Score Groups

**FIGURE 5:**
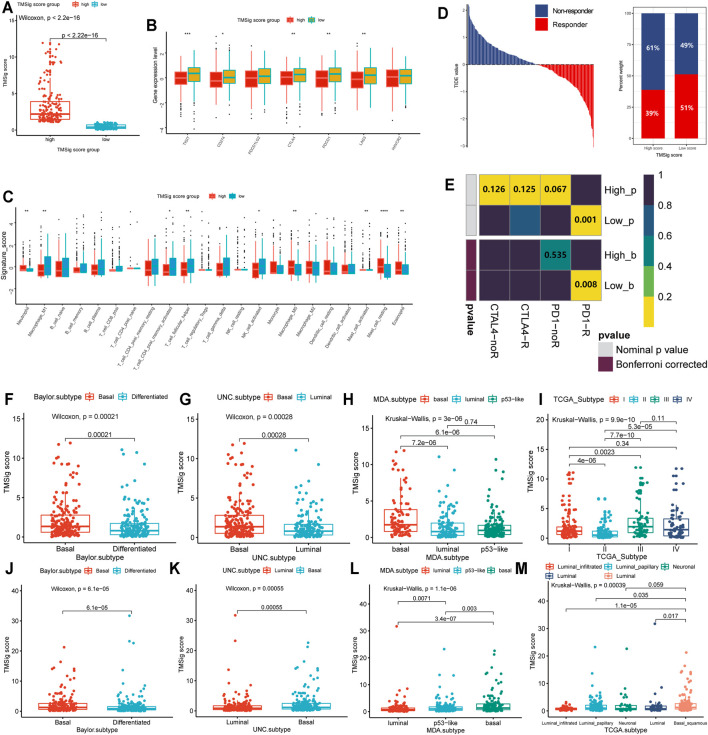
Molecular differences between high- and low-score subgroups and distribution among previous subtypes. **(A)** The TMSig score in the high score group was significantly higher than that in the low score group. **(B)** The expression level of immune checkpoints in the high score and low score groups. **(C)** Immune infiltration cells are expressed in the high score and low score groups (*p < 0.05, **p < 0.01, ***p < 0.001, ****p < 0.0001). **(D)** The immune therapy response and TIDE value of patients with urothelial cancer. **(E)** The subclass mapping analysis showed a significant difference in response to anti-PD-1 therapy among these two groups. The distribution of TMSig score of the IMvigor210 cohort in molecular subtypes. **(F)** Baylor subtype, **(G)** UNC subtype, **(H)** MDA subtype, **(I)** TCGA subtypes. The distribution of TMSig score of the TCGA cohort in molecular subtypes. **(J)** Baylor subtype, **(K)** UNC subtype, **(L)** MDA subtype, **(M)** TCGA subtypes.

The high-score group had a higher TMSig score (Wilcoxon, *p* < 2.2e−16) (Figure 5A), indicating that the two groups have unique features not only in prognosis but also in immune-related characteristics. TIGIT, CD274, CTLA4, PDCD1, and LAG3 presented higher expression levels in the low score group (*p* < 0.05) (Figure 5B), which suggests that people with low TMSig scores might have a better response to immunotherapies targeting immune checkpoints. The immune infiltration cell analysis also showed that these two groups had significantly different marker immune cells (Figure 5C). Then, we used the Tumor Immune Dysfunction and Exclusion (TIDE) algorithm to evaluate each patient’s potential response to immunotherapy and observed that the responsiveness of immunotherapy in the low-score group was higher than that in the high-score group (*p* = 0.02) (Figure 5D). Moreover, subclass mapping was performed with another group of 47 melanoma patients who responded to immunotherapy (Roh et al., 2017). We were encouraged by the observation that a low score indicated potential patient response to PD-1 treatment. (Bonferroni corrected *p* = 0.008) (Figure 5E). These results reconfirmed the application value of the TMSig. To further explore the significantly enriched pathways of the DEGs between the two groups, we carried out GSEA using the clusterProfiler and fgsea R packages. It was found that HALLMARK ANGIOGENESIS, HALLMARK TGF-ß_ SIGNALING, HALLMARK APOPTOSIS, HALLMARK HYPOXIA, and HALLMARK P53 PATHWAY were significantly enriched in the upregulated genes (Supplementary Table S8), which may be related to poor prognosis.

In addition, we found that the high-score samples of the Baylor subtype and UNC subtype were mainly concentrated in the basal type (Wilcoxon, Baylor subtype, *p* = 2.1e-04; UNC subtype, *p* = 2.8e-04) (Figures 5F,G), and the TMSig score in the basal MDA subtype was also significantly higher than that in the luminal type and p53-like type (Kruskal−Wallis, p = 3e−06) (Figure 5H). In the TCGA subtypes, high scores were mainly distributed in clusters III and IV (Figure 5I), and it is generally believed that clusters I/II and III/IV in the molecular subtypes officially obtained by the TCGA are similar to differentiated (or luminal) and basal tumors, respectively (Mo et al., 2018). We also verified the distribution of TMSig scores among the four classification methods in the independent TCGA-BLCA dataset, and the results were consistent with those of the IMvigor210 cohort (Figures 5J–M). Therefore, we inferred that the TMSig is closely related to molecular subtypes of bladder cancer, and different scores may indicate different molecular characteristics, which is of great significance for further understanding the characteristics of these two groups.

### Identification of Potential Therapeutic Agents for High TMSig Score Patients

**FIGURE 6:**
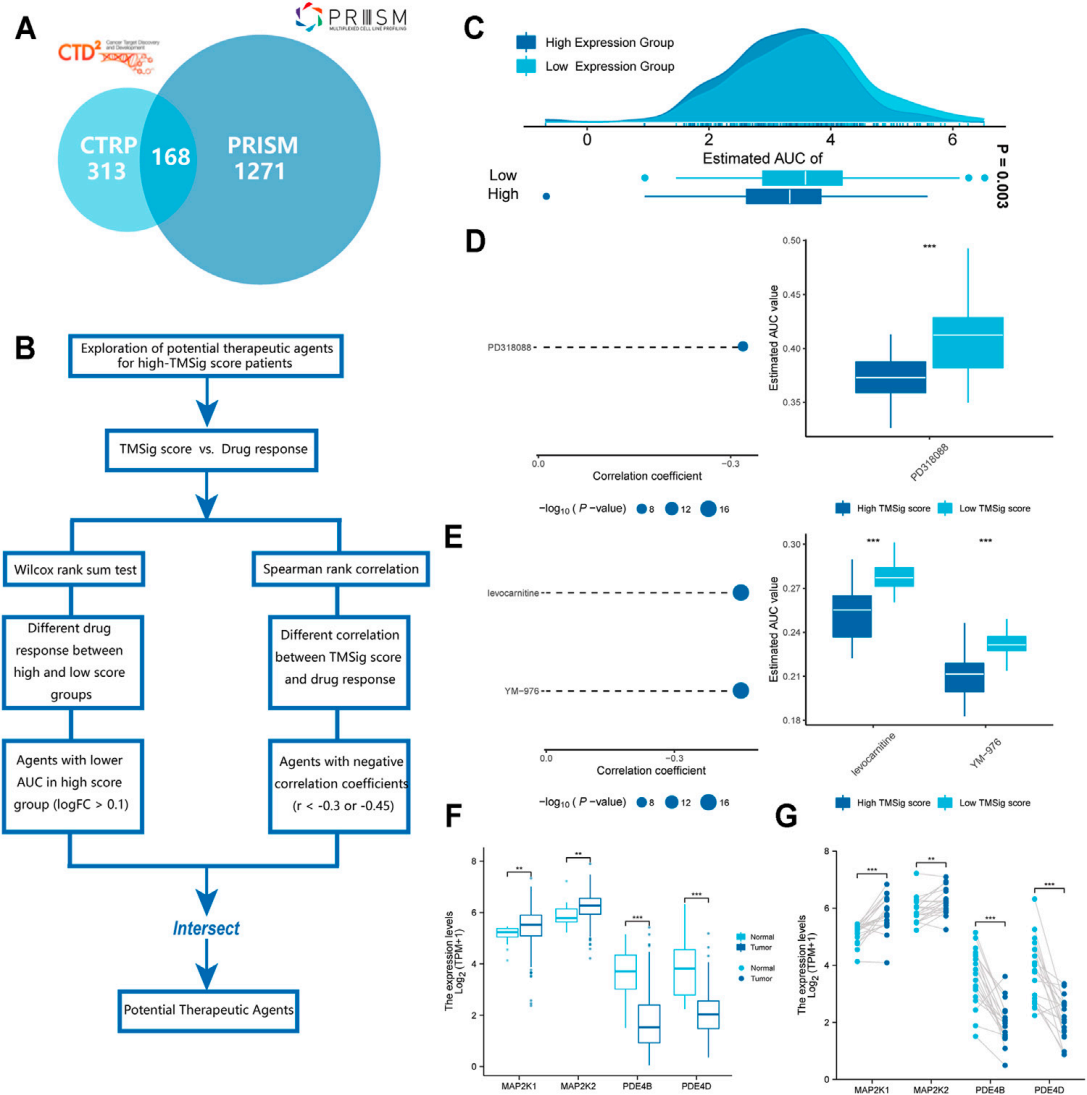
Identification of candidate agents with higher drug sensitivity in high-TMSig score patients. **(A)** A venn diagram of compounds from the CTRP and the PRISM datasets. **(B)** Schematic outlining the strategy for identification of potential therapeutic agents. **(C)** Comparison of estimated cisplatin’s sensitivity (logAUC) between high GULP1 expression and low GULP1 expression groups. **(D)** The results of differential drug response analysis and Spearman’s correlation analysis of compounds from CTRP datasets. **(E)** The results of differential drug response analysis and Spearman’s correlation analysis of compounds from PRISM datasets. **(F)** Unpaired comparative analysis of target genes for potential drugs. **(G)** Paired comparative analysis of target genes for potential drugs.

The CTRP and PRISM datasets contain gene expression profiles and drug sensitivity profiles for hundreds of CCLs and can be used to construct predictive models of drug response. After removing duplicate drugs, these two datasets share 168 compounds, for a total of 1752 compounds (Figure 6A). We removed drugs with deletion values greater than 20% and cell lines derived from haematopoietic and lymphoid tissue. Finally, 680 CCLs for 354 compounds in the CTRP dataset and 480 CCLs for 1285 compounds in the PRISM dataset were used for subsequent analyses. The specific screening process is shown in Figure 6B. Before proceeding further, we first demonstrated that the results of drug response estimation are reliable. Cisplatin is a common therapeutic agent for bladder cancer patients, and a recent study showed that high GULP1 expression enhanced the sensitivity of patients to cisplatin (Teramoto et al., 2021). We divided the patients into high and low expression groups according to the expression level of GULP1. The Wilcoxon rank sum test was used to compare the difference in AUC estimates of cisplatin between the two groups, and the results showed that the AUC estimates were significantly higher (*p* = 0.003) in patients with high GULP1 expression (Figure 6C), consistent with the clinical presentation of cisplatin. After verifying the reliability of the calculation method, we adopted a similar analysis method to Yang et al. (2021). First, differential drug response analysis was performed between the group with high TMSig score (upper decile) and the group with low TMSig score (lower decile) to identify the group with high TMSig score (log2FC > 0.10) with low estimated AUC values. Then, by Spearman correlation analysis between AUC value and TMSig score, compounds with negative correlation coefficients (Spearman’s r < −0.30 for CTRP or <0.45 for PRISM). These analyses yielded one CTRP-derived compound (PD318088) and two Prism-derived compounds (Levocarnitine, YM−976) (Figures 6D,E). Secondly, the fold-change difference of the expression level of candidate drug target genes between tumor tissues and normal tissues (including paired analysis and unpaired analysis) was calculated. A higher fold change value indicated a greater potential of candidate agent for UC treatment (PD318088: MAP2K1, MAP2K2; YM−976: PDE4B, PDE4D) (Figures 6F,G). Finally, we searched at PubMed (https://www.ncbi.nlm.nih.gov/PubMed/) to find evidence of candidate compounds for UC treatment. Overall, PD318088 and YM−976, with relatively sufficient evidence, are considered to be the most promising potential treatment drugs for people with high TMSig score.

## Discussion

Mounting evidence has identified the essential role of the TME in the occurrence and development of UC and the prognosis of patients. However, there is still a lack of comprehensive understanding of the tumor microenvironment of UC. So, we comprehensively analyzed a large cohort of UC patients and constructed the TMSig to comprehensively analyze the tumor microenvironment pattern and predict the survival rate of UC patients and guide more accurate and effective applications of immunotherapy and chemotherapy strategies.

Compared with previous published articles, our study has significant innovation and advantages. Our study not only identified TME patterns in patients with urothelial cancer and established TMSig as a metric to quantify individual patients, but also developed a convenient and practical webpage nomogram, which is more clinically useful than the study by Meng et al. (2021). Meanwhile, the predictive power of TMSig for immunotherapy response has been fully validated by the TIDE algorithm, Submap algorithm, and multiple clinical cohorts receiving immunotherapy. Moreover, potential sensitive drugs have been fully explored with the help of the robust approach. Compared with the study of Meireson et al. (2021), it is a greater improvement in the depth and breadth. In addition, we performed the improved lasso algorithm, which is more advanced in its selection compared to the study of Zhang et al. (2021). Besides, we used multiple omics data such as genomics, transcriptomics to make the analysis more in-depth and complete. Compared with many previous reported signatures (Sun et al., 2021a; Sun et al., 2021b; He et al., 2021; Yan et al., 2021), the predictive power of TMsig is more outstanding. Besides, the TMSig scoring system we constructed can effectively assess the immune profile of patients with urothelial cancer and predict patient prognosis, which we have validated with a sample of 1025 cases. To make the TMSig score better applicable to clinical practice, we included TMSig and IC level in the follow-up analysis and constructed a web-based dynamic nomogram. And the high accuracy and better clinical benefit results of this nomogram was well demonstrated by calibration plots and decision curves. In addition, TMSig can identify potential therapeutic agents for high-risk populations and fully validate them with robust methods to guide clinical precision treatment.

In this study, we identified a TME pattern with a stromal-activation subtype, immune-enriched subtype and immune-suppressive subtype based on unsupervised consensus clustering of immune cell infiltration in the TME. These subtypes are characterized by different immunophenotypes and immune states, which are related to different prognostic outcomes and antitumor immunity levels. Mariathasan et al. found that TGF-β inhibits antitumor immunity by limiting T cell infiltration to shape the tumor microenvironment (Tauriello et al., 2018). Blockade of TGF β signal transduction makes it easy to target tumors with anti-PD-1/PD-L1 checkpoint therapy (Panagi et al., 2020). They also found that TGF-β-blocking antibodies and anti-PD-L1 therapy reduced the transduction of TGF-β signaling in stromal cells and improved the infiltration level of T cells into the center of the tumor, thereby stimulating a strong antitumor immune response and causing tumor regression (Tauriello et al., 2018). Based on these findings, we speculate that the stromal-activation subtype may benefit from a combination of immune checkpoint block drugs and TGF-β blockers (Lan et al., 2018; Ravi et al., 2018). The immune-enriched subtype is similar to the known immunoinflammatory phenotype. This finding supports the potential predictive value of the benefits of immunotherapy. Zhao et al. demonstrated that the immunoinflammatory phenotype of triple-negative breast cancer is characterized by the infiltration of CD8^+^ T cells into the tumor parenchyma (Mariathasan et al., 2018). Job et al. reported that the immune-inflammatory type is characterized by a large level of T lymphocyte infiltration and the activation and upregulation of inflammatory and immune checkpoint pathways. This phenotype is associated with better patient prognosis (Zhao et al., 2020). Our study also revealed that the patients in this subtype had the best prognosis outcomes, which is similar to the results of previous studies.

The TMSig score had the strongest significant negative correlation with M1 macrophages and the strongest significant positive correlation with M0 macrophages. Regarding the low-score group, M1 macrophage infiltration was significant, and the prognosis was good, while in the high-score group, M0 macrophage infiltration was significant, and the prognosis was poor. This suggests that the different states of macrophages may be an important reason for the difference in prognosis among patients with different scores. Tumor-associated macrophages (TAMs) are one of the most abundant matrix components in the tumor microenvironment (Mantovani et al., 2008; Hanahan and Weinberg, 2011; Li et al., 2019b). Previous studies have mainly focused on M2 macrophages because they account for the vast majority of TAMs and have the potential for transformation (Ge et al., 2019). However, M0 and M1 macrophages have attracted increasing attention. M2 macrophages differentiated from M0 macrophages were also highly infiltrated in the population with high infiltration of M0 macrophages, which inhibited inflammation, T cell proliferation and differentiation and promoted angiogenesis of the tumor matrix and tumor cell proliferation (Bingle et al., 2002; Gordon, 2003; Pollard, 2004; Mantovani et al., 2005; Hume, 2015). These mechanisms cannot be ignored due to the poor prognosis of this population. In addition, M1 macrophages have been proven by a previous study to be an important marker for predicting patient prognosis outcomes and the immunotherapy response of patients with mUC (Zeng et al., 2020; Mantovani et al., 2017), and their anticancer ability, such as activating the inflammatory response, participating in host innate immunity and inhibiting tumor cells in the TME, has also been widely recognized (Bingle et al., 2002; Gordon, 2003; Pollard, 2004; Mantovani et al., 2005; Hume, 2015). Samples with high M1 infiltration levels often show immune activation, while those with low M1 infiltration may show an activation of steroid hormone metabolism, which may promote the exclusion of CD8^+^ T cells from the TME (Zeng et al., 2020; Ma et al., 2019). Therefore, the different states of macrophages between the high- and low-score groups worth investigating further and may contribute to the accurate application of treatments (Li et al., 2019b; Tang et al., 2013).

In this study, we found that the high TMSig score group was mainly distributed in the basal subtype, with poor prognosis and significantly lower expression levels of immune checkpoints, which was consistent with previously reported characteristics of the basal subtype (Mo et al., 2018), and the low-score group presented similar characteristics to the differentiated (or luminal) subtype. This indicates that the TMSig score could effectively represent the tumor differentiation status of the samples. In addition, the TMSig score verified the robustness of the prediction of patient immunotherapy response in multiple independent cohorts and was not limited to the comparison of the expression levels of relevant genes, which also demonstrates the superiority of our approach in comparison with signatures reported in previous studies. EGFR pathways are specifically activated in basal-like MIBC. *In vitro* and *in vivo* experiments have also proven that basal-like MIBC cell lines are sensitive to EGFR inhibitors, suggesting that EGFR has great potential as a basal-like MIBC treatment target (Rebouissou et al., 2014). Due to the close correlation between the high-score group and basal-like MIBC, EGFR is worth further investigations in this group. In addition, because of the remarkable tumor heterogeneity in bladder cancer, research on the subtype-specific targets and treatment therapies of bladder cancer is important and urgent.

High TNB and TMB in tumors are related to enhanced responses to immunotherapy (Samstein et al., 2019; Zeng et al., 2020). The new antigens generated by somatic cell mutations in tumors represent a promising method to promote tumor immune recognition. The main hypothesis of immunotherapy is that tumors with elevated TMB will have more new antigens and therefore have higher immunogenicity (Wolf et al., 2019). The TMSig score was closely related to immune response predictors, suggesting that it may be related to different immunotherapy responses. Independent prognostic analysis showed that the TMSig score is an independent prognostic factor for UC patients and is not affected by other factors. The correlation coefficient between them was relatively low, indicating that the TMSig score, TMB, and TNB represent different aspects of tumor immune features. In addition, the high- and low-score groups not only had significant differences in survival and prognosis outcomes but also had significant differences in responses to immunotherapy. GSEA also showed that many carcinogenic pathways were significantly activated in high-score patients. The sensitivity of patients with high and low scores to different chemotherapeutic drugs has also been explored, which will provide new clinical treatment ideas for patients with urothelial carcinoma.

In the current study, we combined multicohort and multigroup data to comprehensively evaluate multidimensional features associated with TME infiltration patterns. We constructed the TMSig, an effective predictor of prognosis, immunotherapy response by scoring patients, which provides new insights into the identification of subtype-specific populations and markers. The low-score group had a better prognosis, better response to immunotherapy, stronger infiltration of M1 macrophages and was more inclined to be in the luminal (differentiated) molecular subtypes. In addition, macrophage-targeted therapy should be considered. Giving full consideration to the antitumor effect of M1 macrophages may have an essential impact on the prognosis of UC patients. Although there is significantly difference of the immune checkpoint distribution between high and low score groups, they had some overlap. So, its clinical application should be more cautious in predicting immune checkpoints. Besides, our findings should be further verified in more prospective cohorts to define the clinical application value more accurately. The important role of macrophages in UC patients should be further explored at the single-cell level. Since not all patients with higher TMSig scores benefit from immunotherapy, more meaningful clinical features should be included in the predictive model to improve its accuracy.

## Conclusion

Through a comprehensive and systematic analysis of the TME characteristics of UC patients, we identified the TMSig score as an independent prognostic factor. The TMSig score can not only accurately predict the prognosis outcomes of patients with UC but also robustly predict patient immunotherapy response in multiple independent cohorts. Interestingly, we found that the TMSig score may play a unique role in high-grade and advanced-stage UC. The high- and low-risk TMSig score groups are in good agreement with the previously recognized molecular subtypes. This enables us to combine histopathological staging with molecular subtypes, comprehensively evaluate the samples, and inspire new ideas for subtype-specific precision therapy. We also found that the difference in the state of macrophages may be the essential factor underlying the difference in patient prognoses. The in-depth study of macrophage-targeted therapy would have great value in advancing the individualized therapy approach for patients with urothelial cancer.

## Data Availability

The original contributions presented in the study are included in the article/Supplementary Material, further inquiries can be directed to the corresponding authors.
