# Supplementary material for: Multi-Omics Analysis of Novel Signature for Immunotherapy Response and Tumor Microenvironment Regulation Patterns in Urothelial Cancer
Source: Front Cell Dev Biol. 2021 Dec 3;9:764125. doi: 10.3389/fcell.2021.764125 (PMC8678486; doi:10.3389/fcell.2021.764125)
Supplement: Supplementary file 2 [file DataSheet2.docx]

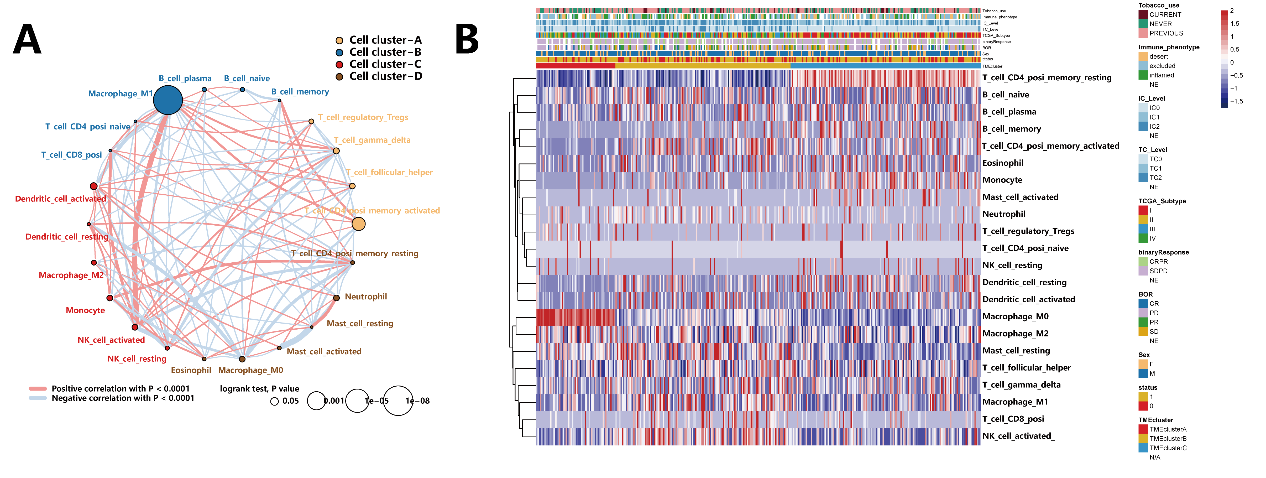


**Figure S1:** (A) Cellular interaction of the TME immune cell types. The size of each cell represents the survival impact of each TME immune cell type, calculation used the formula log10 (log-rank test P values indicated). The lines connecting TME cells represent cellular interactions. The thickness of the line represents the strength of correlation estimated by Spearman correlation analysis. The positive correlation is indicated in red and the negative correlation in blue. (B) Heatmap on the level of immune cell infiltration and clinical information of the IMvigor210 cohort.


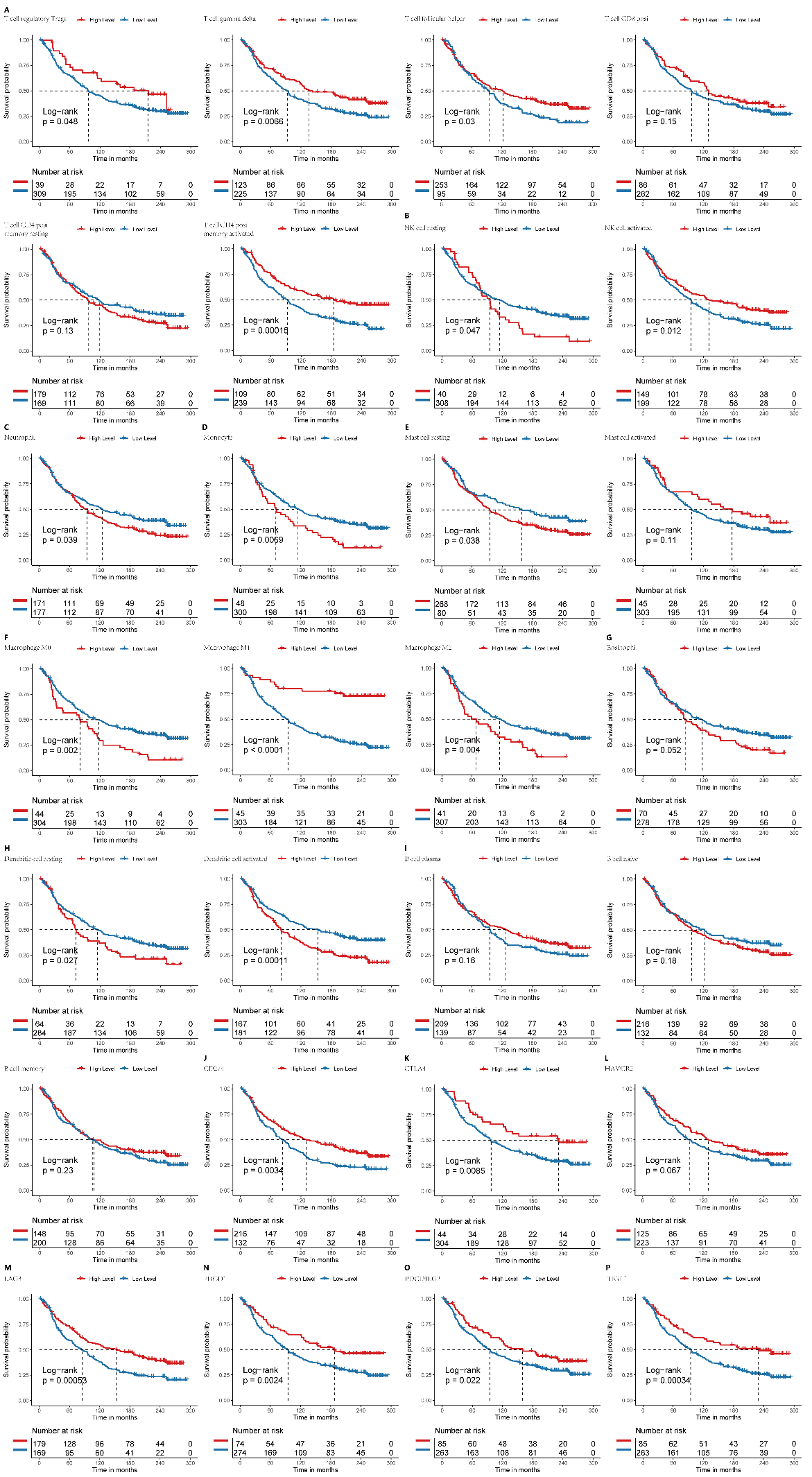


**Figure S2:** Survival analysis of immune cell level and immune checkpoint expression level. (A) T cell-associated cell. (B) NK cell-associated cell. (C) Neutrophil. (D) Monocyte. (E) Mast cell-associated cell. (F) Macrophage-associated cell. (G) Eosinophil. (H) Dendritic cell-associated cell. (I) B cell-associated cell. (J) CD274. (K) CTLA4. (L) HAVCR2. (M) LAG3. (N) PDCD1. (O) PDCD1LG2. (P) TIGIT.


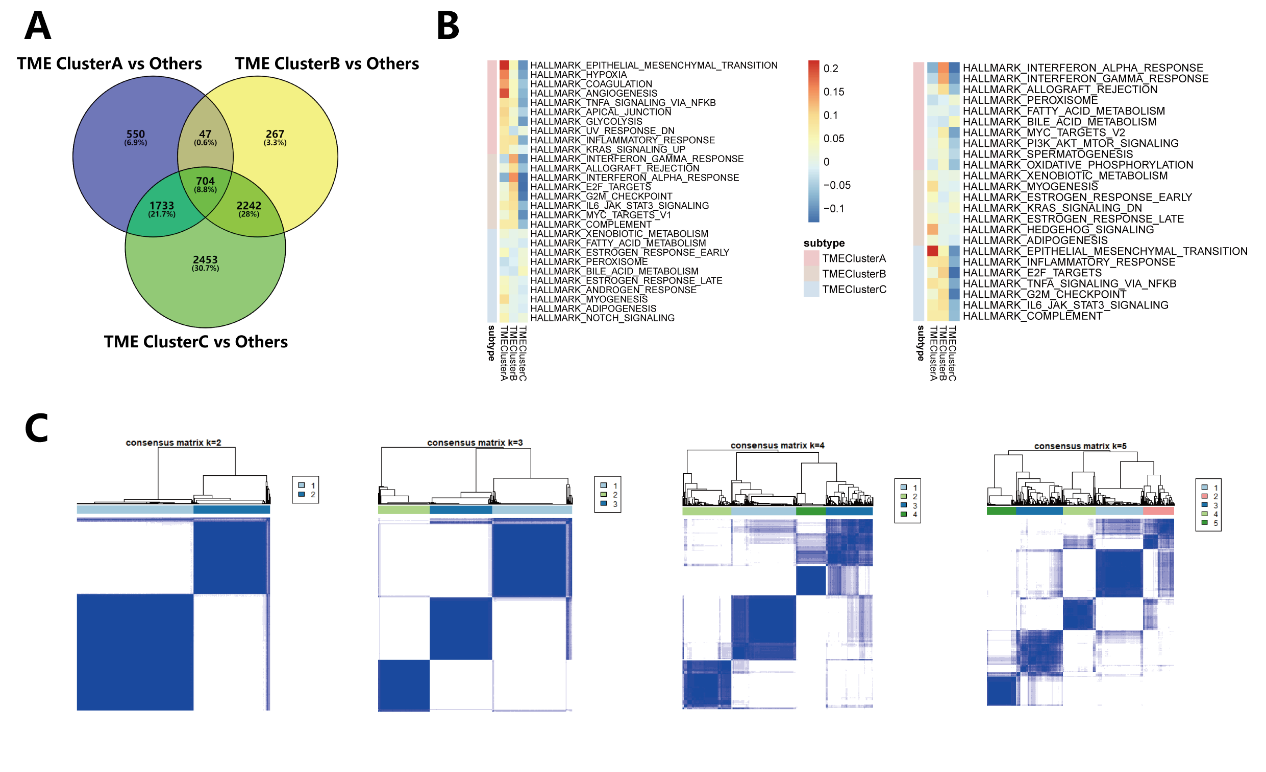


**Figure S3:** (A) Venn diagram illustrating the number of DEGs among TME-Cluster A,

B, and C. (B) GSEA analysis reveals distinct enriched gene sets between clusters. We screened 10 up-regulated pathways (left figure) and 10 down-regulated pathways (right figure) specifically enriched by each cluster. If the same pathway showed the same up-regulation or down-regulation in more than two clusters, the pathway was retained only once. The rows were the gene sets selected and columns were gene clusters. (C) Consensus matrixes of IMvigor210 cohort based on the DEGs expression for each k (k = 2–5), displaying the clustering stability using 1000 iterations of hierarchical clustering.


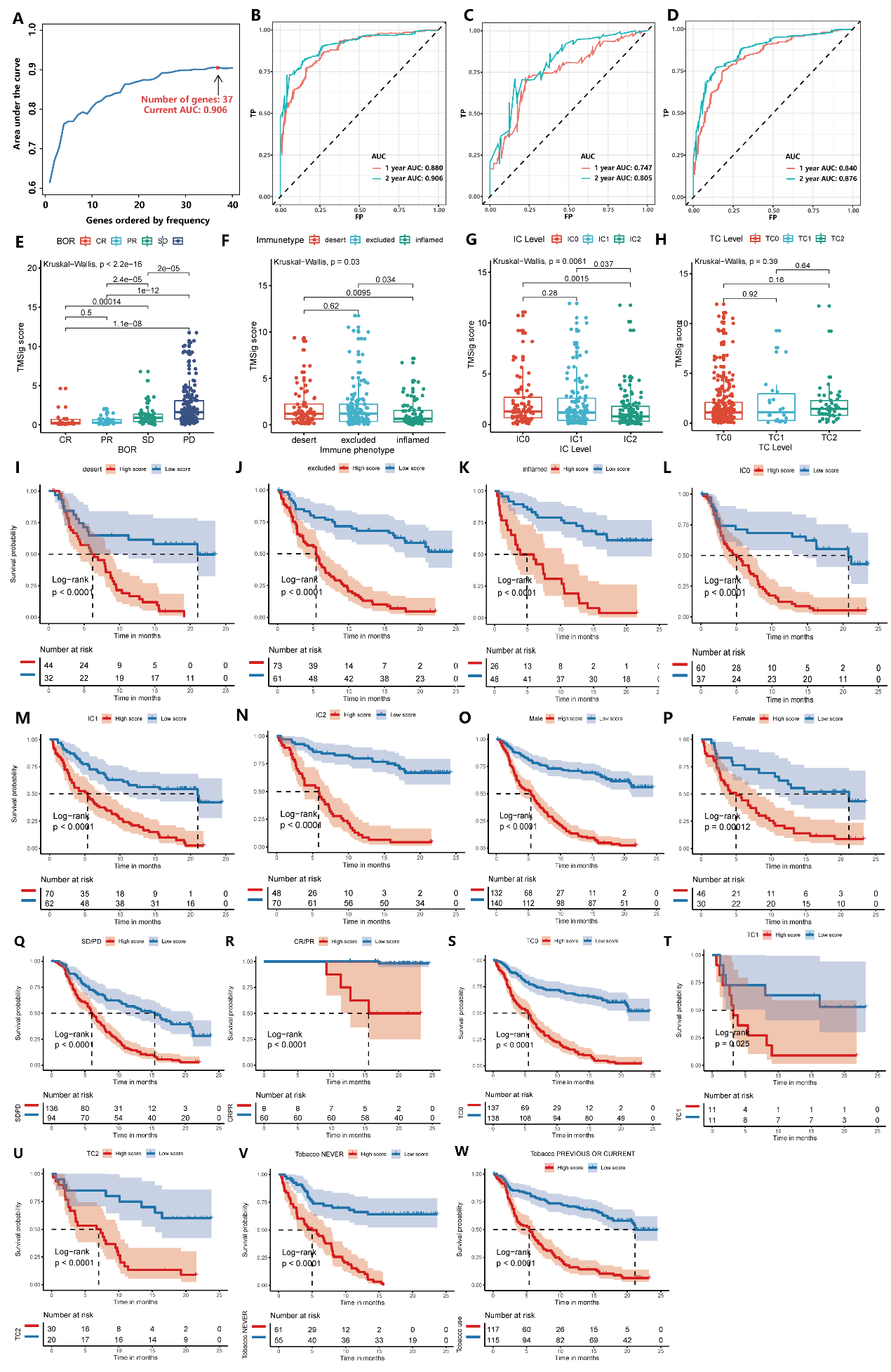


**Figure S4:** (A) The curve of every AUC value generated by ROCs of candidate gene signatures and to identify the highest point of the AUC. (B) The time-dependent ROC curve of UC patients from the training cohort. (C) The time-dependent ROC curve of UC patients from the testing cohort. (D) The time-dependent ROC curve of UC patients from the IMvigor210 cohort. The distribution of TMSig score in various different characters subgroups. (E) BOR: CR, PR, SD, PD. (F) Immunetype: Desert, Excluded, Inflamed. (G) IC level: IC0, IC1, IC2. (H) TC level: TC0, TC1, TC2. The stratified survival Analysis of IMvigor210 cohort, Immunetype: (I)-(K); IC level: (L)-(N); Gender: (O)-(P); BinaryResponse: (Q)-(R); TC level: (S)-(U); Tobacco history: (V)-(W). Log-rank P < 0.05 mean the high and low score group had significant difference in prognosis outcome.


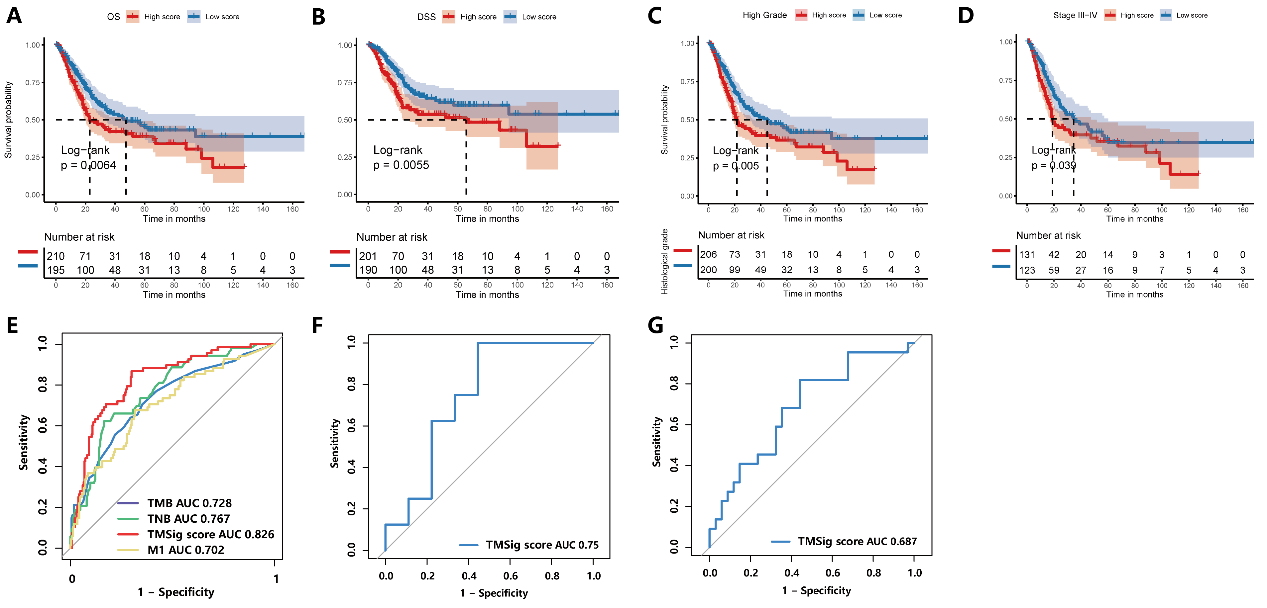


**Figure S5:** The Kaplan-Meier survival Analysis of TCGA cohort, OS: (A); DSS: (B); High Grade: (C); Stage III-IV: (D), Log-rank P < 0.05 mean the high and low score group had significant difference in prognosis outcome. (E) The ROC curve showed the predictive ability of TMSig score, TMB, TNB, and Macrophage M1. (F) Validation of predictive value of TMSig score to immunotherapy in the queue of Miao et al. (G) Validation of predictive value of TMSig score to immunotherapy in GSE35640.
