# Supplementary material for: Multi-Omics Analysis of Novel Signature for Immunotherapy Response and Tumor Microenvironment Regulation Patterns in Urothelial Cancer
Source: Front Cell Dev Biol. 2021 Dec 3;9:764125. doi: 10.3389/fcell.2021.764125 (PMC8678486; doi:10.3389/fcell.2021.764125)
Supplement: Supplementary file 3 [file DataSheet1.docx]

**Multi-Omics Analysis of Novel Signature for Immunotherapy Response and Tumor Microenvironment Regulation Patterns in Urothelial Cancer**

**Supplementary Materials and Methods**

**1. Data source and preprocessing**

Multiomics data of UC patients such as genomic data, transcriptome data, and clinical information, were obtained from http://research-pub.gene.com/IMvigor210CoreBiologies, which is available under the Creative Commons 3.0 license. Data from the TCGA were downloaded from the UCSC Xena browser (<https://gdc.xenahubs.net>). The expression value of FPKM (fragments per kilobase million normalized) was transformed into transcripts per kilobase million (TPM) values to make the data more comparable between sample(1). The survival information of patients from the TCGA was obtained from the study of Liu et al(2). The sample information, updated clinical data, and somatic mutation data for the TCGA-BLCA cohort patients were downloaded from the Genomic Data Commons (https://portal.gdc.cancer.gov/) through the TCGAbiolinks R package(3). The GSE32548 cohort(4) and the GSE48075 cohort(5) were obtained from the Gene Expression Omnibus (GEO) database, and the corresponding clinical information was obtained from the supplementary files of the articles to which they belonged. Samples with incomplete clinical information were removed from the analysis. For the UTUC cohort, we downloaded the raw data from the BioProject (PRJNA678814), used fastqc to quality control the raw data, and used trim-galore for filtering. Then the data were subsequently compared through hisat2 with the gencode27 version of the genome. Next, we usd featureCounts to quantify the bam files, and filter the low-expressed genes according to the criteria of Su et al(6). The clinical information was obtained from the supplementary files reported by Su et al(6). And then we merged the individual datasets by the robust Combat algorithm to eliminate batch effects among the datasets(7), and the merged data were examined by a PCA algorithm.

**2. Estimation of immune cell infiltration in TME**

The CIBERSORT algorithm(8) and LM22 gene signature were used to quantify the proportion of immune cells in urothelial cancer. CIBERSORT is a deconvolution algorithm based on support vector regression that uses a set of minimum reference gene expression values corresponding to each cell type to infer the proportion of cell types in large tumor sample data of mixed cell types. CIBERSORT uses the empirical p-value of Monte Carlo sampling to calculate deconvolution to indicate the accuracy of the results. P < 0.05 indicates that the inferred cell composition is high and reliable. The immune cell infiltration level calculated by the CIBERSORT algorithm in the IMvigor210 cohort was obtained from the supplementary files of the published articles from Zeng et al(9).

**3. Consensus clustering to determine the pattern of immune cell infiltration in the TME**

We used the " ConsensusClusterPlus " R package(10) for unsupervised clustering to determine the different TME cell infiltration patterns. “Euclidean” was selected as the distance, and the clustering process was repeated 1000 times to ensure the stability of the results. The PCA algorithm(9, 11) was performed to calculate the score of each sample on other signatures related to the tumor microenvironment(12, 13).

**4. Identification of differentially expressed genes related to TME patterns**

Patients were divided into different groups based on the level of immune cell infiltration. The gene expression data of patients in each TME cluster were compared with those of other patients using the limma package(14) to obtain DEGs related to TME clusters. TME cluster-related DEGs were defined with adjusted P < 0.05.

**5. Biological function and pathway enrichment analysis**

The R package “clusterProfiler”(15) was used to analyze the functional enrichment of DEGs among different TME clusters, and GO terms were identified through the strict cutoff of q >0.05 and p < 0.05. In addition, we also analyzed the pathway of specific enrichment among TME clusters by the “GSVA” R package(16). The “h.all.v7.1.symbols.gmt” gene set was obtained from MSigDB of the Broad Institute for running GSVA analysis.

**6. Feature selection and Construction of TME gene signature**

To evaluate the TME patterns of an individual tumor, we generated a scoring system, the TME signature, and defined the calculated score as the TMSig score. The specific construction process is as follows:

First, we used DEGs between TME clusters for unsupervised clustering and randomly divided patients in the IMvigor210 cohort into the training set and the testing set (7:3) for further study. Then, we performed a univariate Cox regression analysis to select prognosis-related DEGs. We defined the genes with P < 0.01 as TME metagenes significantly related to prognosis and included them in the next step. Next, similar to that performed in previous studies(17, 18), we performed improved Lasso regression with 10-fold cross validation. The Lasso regression cycle ran 1000 times, each cycle was randomly stimulated 1000 times, and the frequency of each gene included in the 1000 repetitive Lasso regression model was recorded. Genes with a frequency over 500 were defined as key genes. These genes were evaluated by Cox proportional hazard regression analysis, and the signature was constructed. The Area Under Curve (AUC) values of each model were recorded and drawn as AUC curves. When the curve reached the highest point, the AUC value of the model was maximum, the calculation process was terminated, and the model obtained at this time was regarded as the best candidate model. The calculation formula was as following:

$$TMSig score=\sum_{i=1}^{n} Coef i *Expr i$$

Coef i refers to the coefficient of TME-related gene i; “Expr i" is the expression value of the selected gene from the signature. We calculated and summed the result of each gene in TMSig, and the sum was the TMSig score for each sample.

**7. Gene expression and clinical data of multiple cohorts treated with immunotherapy**

The transcriptomic data and clinical information of three cohorts from patients with metastatic urothelial cancer who received the anti-PD-L1 agent, patients with metastatic melanoma and non-small-cell lung cancer who received MAGE-3 agent-based treatment(19), and patients with metastatic clear cell renal cell carcinoma who received anti-PD-1 monotherapy(20) were downloaded to verify the accuracy and application potential of the TMSig for predicting the immunotherapy response of patients. For the metastatic urothelial cancer cohort, the count data were normalized by the trimmed mean of M-values and transformed with voom to log2-counts per million with associated precision weights(14). For the metastatic ccRCC cohort treated with anti-PD-1 monotherapy, the gene expression for all patient samples (TPM normalization) was downloaded from the supplementary file of Miao et al(20). For the GSE35640 cohort (N = 55), the data were downloaded from the GEO database.

**8. Exploration of the potential of the** **TMSig score in clinical application**

To further evaluate the value of the TMSig score in clinical application, we performed univariate and multivariate Cox regression analysis with several important clinical features to verify its prognostic role for UC patients. The difference in clinical response to immunotherapy between high- and low-risk groups was verified and analyzed again by using TIDE (21) and subclass mapping algorithms (22). Meanwhile, based on “BLCAsubtyping” (23), we explored the association between the TMSig score and molecular subtypes of bladder cancer identified by previous studies. We also explored the differences in molecular characteristics between high- and low-score groups, which could have important reference value for the identification of high-risk populations and the selection of precise therapies.

**9. Screening for potential therapeutic agents**

Expression profile data for human cancer cell lines (CCL) data was from the Broad Institute Cancer Cell Line Encyclopedia (CCLE)(24). Drug sensitivity data for CCLs were obtained from CTRP v.2.0 (https://portals.broadinstitute.org/ctrp) and PRISM Repurposing dataset (19Q4, https://depmap.org/portal/prism/). Both datasets provide area under the dose response curve (AUC) values as a measure of drug sensitivity, and lower AUC values mean increased sensitivity to treatment.

**10. Statistical analysis**

For comparisons of two groups, the normally distributed variables were tested by unpaired Student’s t-tests, and the nonnormally distributed variables were tested by the Wilcoxon rank-sum test. For comparisons of more than two groups, the parametric and nonparametric variables were tested by one-way ANOVA tests and Kruskal-Wallis tests, respectively(25). The two-sided Fisher’s exact test was performed for the contingency tables. According to the relationship between the TMSig score and the overall survival rate of the patients, we determined the cutoff value of the TMSig score through the “surv-cutpoint” function of the “survminer” R package. We repeatedly tested all possible cut-off points to identify the maximum rank statistics and then used the two-classification method to classify the TMSig score. According to the maximum selected logarithmic rank statistics, patients could be distinguished into high- and low-score groups in each cohort to reduce the computational batch effect, which is similar to that reported by Zeng et al(11), Zhang et al(26) and Chong et al(27). The survival curves of each data set were generated by Kaplan-Meier analysis, and statistically significant differences were determined through the log-rank test. The ROC curve was used to assess the specificity and sensitivity of the TMSig score, and the AUC was quantified by the “pROC” package(28). The mutation landscape of patients in the TCGA-BLCA cohort was depicted through the maftools R package(29). All statistical p values were two-sided, and p < 0.05 was considered to be statistically significant. All data processing was performed using R 3.6.1.

**Reference:**

1. Wagner GP, Kin K, Lynch VJ. Measurement of mRNA abundance using RNA-seq data: RPKM measure is inconsistent among samples. *Theory Biosci* (2012) 131(4):281-5. doi: 10.1007/s12064-012-0162-3. PubMed PMID: 22872506.

2. Liu J, Lichtenberg T, Hoadley KA, Poisson LM, Lazar AJ, Cherniack AD, et al. An Integrated TCGA Pan-Cancer Clinical Data Resource to Drive High-Quality Survival Outcome Analytics. *Cell* (2018) 173(2). doi: 10.1016/j.cell.2018.02.052. PubMed PMID: 29625055.

3. Colaprico A, Silva TC, Olsen C, Garofano L, Cava C, Garolini D, et al. TCGAbiolinks: an R/Bioconductor package for integrative analysis of TCGA data. *Nucleic Acids Res* (2016) 44(8):e71. doi: 10.1093/nar/gkv1507. PubMed PMID: 26704973.

4. Lindgren D, Sjödahl G, Lauss M, Staaf J, Chebil G, Lövgren K, et al. Integrated genomic and gene expression profiling identifies two major genomic circuits in urothelial carcinoma. *PLoS One* (2012) 7(6):e38863. doi: 10.1371/journal.pone.0038863. PubMed PMID: 22685613.

5. Choi W, Porten S, Kim S, Willis D, Plimack ER, Hoffman-Censits J, et al. Identification of distinct basal and luminal subtypes of muscle-invasive bladder cancer with different sensitivities to frontline chemotherapy. *Cancer Cell* (2014) 25(2):152-65. doi: 10.1016/j.ccr.2014.01.009. PubMed PMID: 24525232.

6. Su X, Lu X, Bazai SK, Compérat E, Mouawad R, Yao H, et al. Comprehensive integrative profiling of upper tract urothelial carcinomas. *Genome Biol* (2021) 22(1):7. doi: 10.1186/s13059-020-02230-w. PubMed PMID: 33397444.

7. Johnson WE, Li C, Rabinovic A. Adjusting batch effects in microarray expression data using empirical Bayes methods. *Biostatistics* (2007) 8(1):118-27. PubMed PMID: 16632515.

8. Newman AM, Liu CL, Green MR, Gentles AJ, Feng W, Xu Y, et al. Robust enumeration of cell subsets from tissue expression profiles. *Nat Methods* (2015) 12(5):453-7. doi: 10.1038/nmeth.3337. PubMed PMID: 25822800.

9. Zeng D, Ye Z, Wu J, Zhou R, Fan X, Wang G, et al. Macrophage correlates with immunophenotype and predicts anti-PD-L1 response of urothelial cancer. *Theranostics* (2020) 10(15):7002-14. doi: 10.7150/thno.46176. PubMed PMID: 32550918.

10. Monti S, Tamayo P, Mesirov J, Golub T. Consensus Clustering: A Resampling-Based Method for Class Discovery and Visualization of Gene Expression Microarray Data. *Machine Learning* (2003) 52(1):91-118. doi: 10.1023/A:1023949509487.

11. Zeng D, Li M, Zhou R, Zhang J, Sun H, Shi M, et al. Tumor Microenvironment Characterization in Gastric Cancer Identifies Prognostic and Immunotherapeutically Relevant Gene Signatures. *Cancer immunology research* (2019) 7(5):737-50. doi: 10.1158/2326-6066.CIR-18-0436. PubMed PMID: 30842092.

12. Li Y, Jiang T, Zhou W, Li J, Li X, Wang Q, et al. Pan-cancer characterization of immune-related lncRNAs identifies potential oncogenic biomarkers. *Nat Commun* (2020) 11(1):1000. doi: 10.1038/s41467-020-14802-2. PubMed PMID: 32081859.

13. Mariathasan S, Turley SJ, Nickles D, Castiglioni A, Yuen K, Wang Y, et al. TGFβ attenuates tumour response to PD-L1 blockade by contributing to exclusion of T cells. *Nature* (2018) 554(7693):544-8. doi: 10.1038/nature25501. PubMed PMID: 29443960.

14. Ritchie ME, Phipson B, Wu D, Hu Y, Law CW, Shi W, et al. limma powers differential expression analyses for RNA-sequencing and microarray studies. *Nucleic acids research* (2015) 43(7):e47. doi: 10.1093/nar/gkv007. PubMed PMID: 25605792.

15. Yu G, Wang L-G, Han Y, He Q-Y. clusterProfiler: an R package for comparing biological themes among gene clusters. *Omics : a journal of integrative biology* (2012) 16(5):284-7. doi: 10.1089/omi.2011.0118. PubMed PMID: 22455463.

16. Hänzelmann S, Castelo R, Guinney J. GSVA: gene set variation analysis for microarray and RNA-seq data. *BMC Bioinformatics* (2013) 14:7. doi: 10.1186/1471-2105-14-7. PubMed PMID: 23323831.

17. Hong W, Liang L, Gu Y, Qi Z, Qiu H, Yang X, et al. Immune-Related lncRNA to Construct Novel Signature and Predict the Immune Landscape of Human Hepatocellular Carcinoma. *Mol Ther Nucleic Acids* (2020) 22:937-47. doi: 10.1016/j.omtn.2020.10.002. PubMed PMID: 33251044.

18. Sveen A, Ågesen TH, Nesbakken A, Meling GI, Rognum TO, Liestøl K, et al. ColoGuidePro: a prognostic 7-gene expression signature for stage III colorectal cancer patients. *Clin Cancer Res* (2012) 18(21):6001-10. doi: 10.1158/1078-0432.CCR-11-3302. PubMed PMID: 22991413.

19. Ulloa-Montoya F, Louahed J, Dizier B, Gruselle O, Spiessens B, Lehmann FF, et al. Predictive gene signature in MAGE-A3 antigen-specific cancer immunotherapy. *J Clin Oncol* (2013) 31(19):2388-95. doi: 10.1200/JCO.2012.44.3762. PubMed PMID: 23715562.

20. Miao D, Margolis CA, Gao W, Voss MH, Li W, Martini DJ, et al. Genomic correlates of response to immune checkpoint therapies in clear cell renal cell carcinoma. *Science* (2018) 359(6377):801-6. doi: 10.1126/science.aan5951. PubMed PMID: 29301960.

21. Jiang P, Gu S, Pan D, Fu J, Sahu A, Hu X, et al. Signatures of T cell dysfunction and exclusion predict cancer immunotherapy response. *Nature medicine* (2018) 24(10):1550-8. doi: 10.1038/s41591-018-0136-1. PubMed PMID: 30127393.

22. Hoshida Y, Brunet J-P, Tamayo P, Golub TR, Mesirov JP. Subclass mapping: identifying common subtypes in independent disease data sets. *PLoS One* (2007) 2(11):e1195-e. doi: 10.1371/journal.pone.0001195. PubMed PMID: 18030330.

23. Kamoun A, de Reyniès A, Allory Y, Sjödahl G, Robertson AG, Seiler R, et al. A Consensus Molecular Classification of Muscle-invasive Bladder Cancer. *European urology* (2020) 77(4):420-33. doi: 10.1016/j.eururo.2019.09.006. PubMed PMID: 31563503.

24. Ghandi M, Huang FW, Jané-Valbuena J, Kryukov GV, Lo CC, McDonald ER, et al. Next-generation characterization of the Cancer Cell Line Encyclopedia. *Nature* (2019) 569(7757):503-8. doi: 10.1038/s41586-019-1186-3. PubMed PMID: 31068700.

25. Hazra A, Gogtay N. Biostatistics Series Module 3: Comparing Groups: Numerical Variables. *Indian J Dermatol* (2016) 61(3):251-60. doi: 10.4103/0019-5154.182416. PubMed PMID: 27293244.

26. Zhang B, Wu Q, Li B, Wang D, Wang L, Zhou YL. mA regulator-mediated methylation modification patterns and tumor microenvironment infiltration characterization in gastric cancer. *Mol Cancer* (2020) 19(1):53. doi: 10.1186/s12943-020-01170-0. PubMed PMID: 32164750.

27. Chong W, Shang L, Liu J, Fang Z, Du F, Wu H, et al. mA regulator-based methylation modification patterns characterized by distinct tumor microenvironment immune profiles in colon cancer. *Theranostics* (2021) 11(5):2201-17. doi: 10.7150/thno.52717. PubMed PMID: 33500720.

28. Robin X, Turck N, Hainard A, Tiberti N, Lisacek F, Sanchez J-C, et al. pROC: an open-source package for R and S+ to analyze and compare ROC curves. *BMC Bioinformatics* (2011) 12:77. doi: 10.1186/1471-2105-12-77. PubMed PMID: 21414208.

29. Mayakonda A, Lin D-C, Assenov Y, Plass C, Koeffler HP. Maftools: efficient and comprehensive analysis of somatic variants in cancer. *Genome Res* (2018) 28(11):1747-56. Epub 2018/10/19. doi: 10.1101/gr.239244.118. PubMed PMID: 30341162.
